# Detection and antibiotic resistance of Salmonella isolates from selected poultry farms in Dar es Salaam, Tanzania

**DOI:** 10.1099/acmi.0.000879.v5

**Published:** 2025-05-21

**Authors:** Saxon J. Mwambene, Adelard B. Mtenga, Augustino A. Chengula, Abubakar S. Hoza

**Affiliations:** 1 Tanzania Medicines and Medical Devices Authority, P.O. Box 77150, DSM, Tanzania; 2 Department of Veterinary Microbiology, Parasitology and Biotechnology, College of Veterinary Medicine and Biomedical Sciences, Sokoine University of Agriculture, P.O. Box 3019, Morogoro, Tanzania

**Keywords:** antibiotic resistant, Dar es Salaam, *InvA *gene, multidrug resistant, poultry farms, poultry droppings, *Salmonella *spp.

## Abstract

**Introduction.** *Salmonella* is one of the most prevalent foodborne bacteria, posing a significant global health concern and responsible for ~155,000 deaths and 93.8 million human foodborne illnesses annually. The rampant use of antibiotic agents to combat salmonellosis in poultry has contributed to the emergence of resistance against commonly used antibiotics.

**Methodology.** The cross-sectional study was conducted between January and June 2023 across three districts in the Dar es Salaam region, Tanzania. *Salmonella* isolates were detected and confirmed by using standard microbiological conventional methods and molecular methods such as PCR and genomic sequencing. PCR was used for detecting the presence of the *invA* gene, and partial DNA sequencing was performed to identify species and their close relatedness. A Kirby–Bauer disc diffusion method was employed to evaluate *Salmonella* sensitivity to seven different commonly used antibiotics, namely ampicillin, azithromycin, chloramphenicol, ciprofloxacin, gentamycin, trimethoprim/sulfamethoxazole and tetracycline. *Salmonella* strain with reference number ATCC 8739 was used as a control.

**Results.** The overall *Salmonella* isolates from faecal droppings were 6.04% (*n*=796). Of the PCR-confirmed isolates, 64.3% (*n*=28) were resistant to more than two classes of antibiotics and hence considered multidrug resistant. The highest resistance was observed with ampicillin (92.9%, *n*=26), followed by tetracycline (71.43%, *n*=20), ciprofloxacin (42.9%, *n*=12), sulphonamide (42.85%, *n*=12), gentamicin (35.7%, *n*=10) and azithromycin (28.9%, *n*=8). All the isolates were susceptible to chloramphenicol (100%, *n*=28). Twenty-eight isolates were sent for sequencing, out of which 16 sequences (OR021717-OR021739) met the criteria for phylogenetic analysis. All 16 sequences had a per cent identity to EU348369 strains Senftenberg isolated from China, OL581594 *Salmonella* Newport isolated from China and EU348368 *Salmonella* Pullorum isolates from China. Other sequences diverged more distantly; these are *Salmonella* Abony with accession number CP007541 and *Salmonella* Kentucky with accession number OL581592. The tree also included an outgroup species, *Salmonella bongori*, which was downloaded from GenBank with accession numbers NC015761 and NC021870 *S. bongori*.

**Conclusion.** The high level of antibiotic resistance found in this study could be due to the misuse of antibiotics in poultry management, and/or, probably, there were circulating resistant *Salmonella* strains in the environment. To reverse the trend observed, immediate interventions such as advocating for the prudent use of antibiotics in poultry production systems by strengthening extension services to poultry farmers and the use of a farmer field school model to improve poultry management through improved farm biosecurity are required.

## Data summary

The authors confirm that all supporting data, protocols, software and supplementary materials used in this study are provided either within the manuscript or through publicly accessible repositories. The following resources and tools were employed:

### Genetic sequences

Sixteen *Salmonella invA* gene sequences generated in this study have been deposited in the National Center for Biotechnology Information (NCBI) GenBank repository under accession numbers OR021717 to OR021739. Comparative sequences used for phylogenetic analysis were retrieved from the NCBI GenBank database for reference. All the retrieved individual accession numbers can be found in Table S1, available in the online Supplementary Material.

### Software and analytical tools

BioEdit Software (version 7.7): Used for editing raw sequences and assembling consensus sequences.

mega11 (Molecular Evolutionary Genetics Analysis): Utilized for sequence alignment and phylogenetic tree construction. DOI: 10.1093/molbev/msab120.

SPSS (version 20): Applied for statistical analysis of social demographic data. IBM SPSS Software

GraphPad Prism (version 9.0): Used for generating statistical analyses and graphical representations. DOI: 10.1038/s41592-020-0820-9.

### Antimicrobial susceptibility data

Data on *Salmonella* resistance patterns to seven classes of antibiotics were obtained using the Kirby–Bauer disc diffusion method and interpreted according to the [1] guidelines. Results are detailed within the manuscript and supplementary materials.

### Data accessibility

Supplementary materials, including PCR gel electrophoresis images, antimicrobial susceptibility data and detailed phylogenetic tree files, are included as part of this submission.

### Repositories and links

The NCBI blast tool was used for sequence similarity searches and validation (NCBI blast).

WHO reports and relevant bacteriological guidelines used for contextualizing findings are publicly accessible (WHO AMR Surveillance).

## Introduction

### Background information

*Salmonella* is among the most common foodborne pathogens around the world, which causes about 93.8 million foodborne infections and 155,000 fatalities every year, making it a public health concern globally [2]. This bacterium is highly infective because it contains a protein called *invA*, which is used for the invasion of host epithelial cells [3]. One of the key players in the process of *Salmonella*’*s* intracellular pathogenicity is enhanced by the *invA* gene. The gene helps *Salmonella* to break into the cell and survive in the cell, making the start of its infection cycle.

More than half of the over 2,500 *Salmonella* serotypes found to date are the primary cause of salmonellosis in poultry belonging to *Salmonella enterica* subsp. *enterica* [4]. Globally, the use of poultry meat and eggs has increased due to an increased demand for protein and a desire for high-protein and low-fat diets [5]. This increases the risk of humans being infected with *Salmonella* by eating contaminated poultry products.

In Tanzania, the recent economic growth and the expansion of the middle class led to increased demand for the consumption of poultry meat and products [6]. Although this is good for the poultry industry, it came at a cost. As more people engage in intensive poultry farming, frequent poultry disease outbreaks are justifiable, and salmonellosis is among them. To manage the diseases, farmers use antibiotics not just for treatment but also for growth promotion and prevention of diseases [7,9]. Unfortunately, this type of poultry management has led to extensive antibiotic use, resulting in a substantial increase in *Salmonella* strains resistant to multiple antibiotics over the decades [10].

In Tanzania, contamination with *Salmonella*-resistant strains in the poultry products supply chain, such as meat, meat products and eggs, has been previously reported [11]. The emergence of multidrug-resistant (MDR) *Salmonella* strains impacts both the efficacy of antibiotic therapy and production cost among poultry keepers; this also poses a threat to the poultry industry and public health locally and globally [12]. To ensure safe poultry production, controlling *Salmonella* spp. in poultry farms is essential for food safety and halting the spread of antibiotic-resistant strains [13]. Recognizing this threat, the World Health Organization has defined *Salmonella* as a ‘priority pathogen’, aiming at guiding and promoting further research and development into new antibiotics for its management [14,15].

This study was, therefore, designed to detect the presence of *Salmonella* from poultry faecal droppings and to evaluate the antimicrobial susceptibility profile of *Salmonella* isolates in Dar Es Salaam, Tanzania.

## Methods

### Study location

**Fig. 1.**
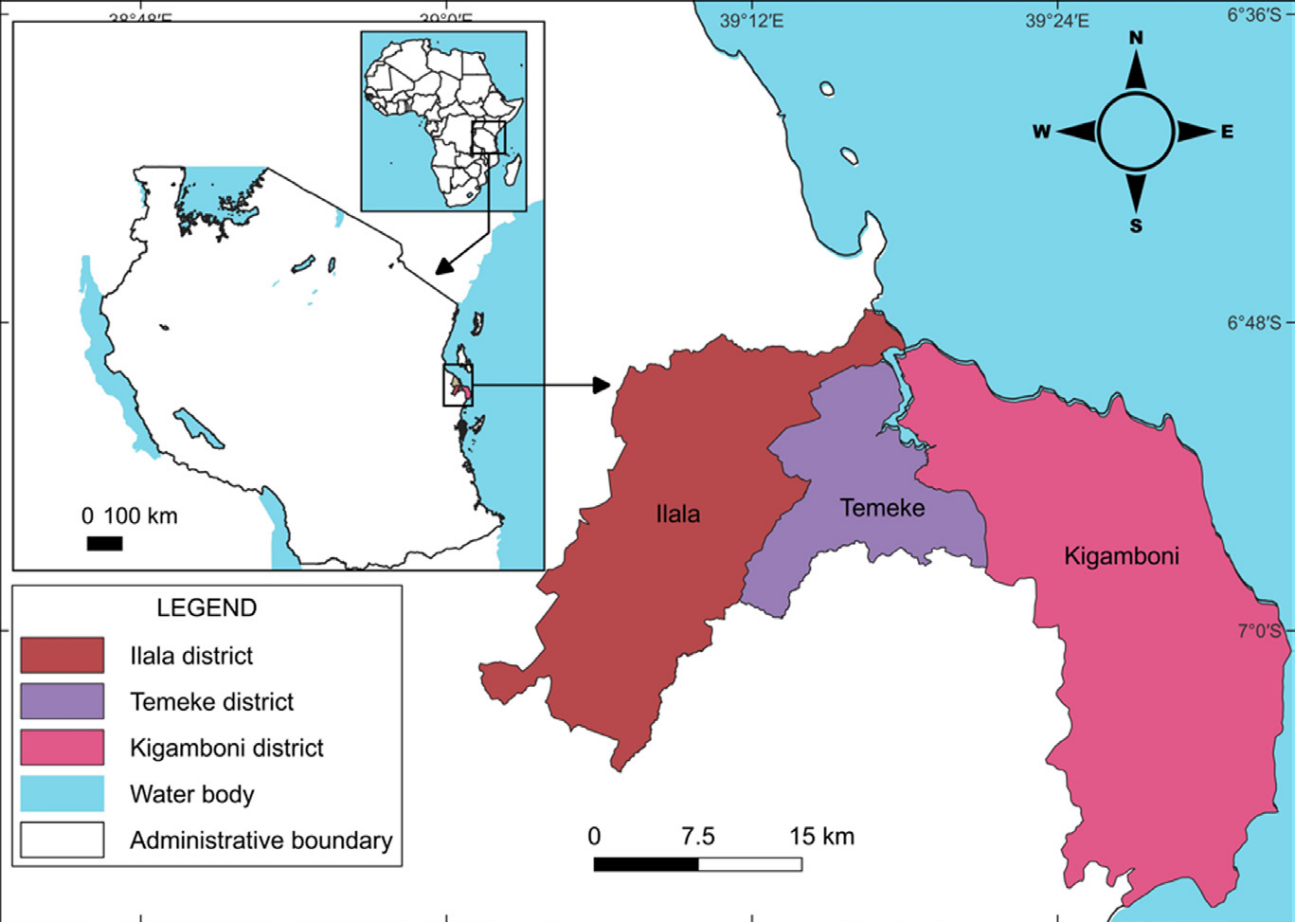
A map showing three districts where the study was conducted.

This study was conducted between January and June 2023 in three districts in Dar es Salaam city (Fig. 1), namely Kigamboni (6.8227°S and 39.3024°E), Temeke (6.9488°S and 39.4450°E) and Ilala (6.9276°S and 39.1336°E).

### Study design and sample size determination

A cross-sectional study design was conducted using a snowball sampling method, whereby each poultry keeper referred us to the location of another poultry keeper. Two hundred twenty-four farmers were identified for sampling. Three samples were collected from each farmhouse. The collection points were at the centre, at the side and around the feeders. Each poultry house yielded three samples, but the sampling strategy was adjusted depending on the structure and size of the farm. If the farm had multiple poultry houses, each house contributed three samples. Therefore, the approach resulted in the collection of 794 faecal samples across 224 farms. During analysis, each sample was used and processed for the isolation of *Salmonella* spp.

### Faecal sample collection

Approximately 10 g of fresh faecal droppings were collected using a sterile spatula from three locations within each poultry house (centre, sides and around the feeders) into sterile containers. Social demographic information of the farmers, including age, sex, marital status, district and level of education, was recorded using sample collection forms. Depending on the size and number of poultry houses, three to five samples were collected at a particular poultry house. Collected faecal samples were then transported in a cold chain to the laboratory at the Tanzania Medicines and Medical Devices Authority (TMDA) in Dar es Salaam. Processing of samples collected was done as described by Orji *et al*. [16].

### Isolation of *Salmonella* spp.

*Salmonella* species were isolated from poultry faecal samples following the method previously described by Paul *et al*. [17]. Briefly, a pre-enrichment process involving weighing ~10 g of faeces using a digital balance, followed by adding 90 ml of buffered peptone water (BPW). The mixture was then vigorously homogenized and incubated at 37 °C for 18–24 h to increase the chance of isolating *Salmonella* spp. Following the overnight culture in BPW, 1 ml of the solution was transferred into 9 ml of Rappaport Vassiliadis medium (RVS) broth and incubated in a water bath at 42 °C for 18–24 h to recover the damaged or stressed cells of *Salmonella* spp. Subsequently, a sterile wire loop full of homogenized samples was cultured on xylose lysine deoxycholate (XLD) agar and incubated for a further 18–24 h at 37 °C.

Colonies exhibiting characteristics of *Salmonella* morphology – bull’s eye-shaped with a typical black centre and smooth greenish periphery appearing on the XLD agar plates after 18–24 h of incubation were identified.

### Biochemical identification of *Salmonella* isolates

The presumptive *Salmonella* colonies were characterized biochemically as described in ISO/TS 6579-2:2012(E). Biochemical identification of probable *Salmonella* colonies was conducted using a series of tests, including the triple sugar iron (TSI) test, urea agar, l-lysine decarboxylation media, and sulphide indole motility (SIM) media. The colonies were incubated at 37 °C for 18–24 h following the method outlined in ISO/TS 6579-2:2012(E). The interpretation of the biochemical tests, typical reactions of most *Salmonella* serovars and percentages of possibilities were described according to ISO/TS 6579-2:2012(E). Finally, the presumptive *Salmonella* isolates were transported to the Genome Science Center Laboratory at the Sokoine University of Agriculture for the detection of the *invA* gene using PCR.

### Molecular identification of *Salmonella* spp*.*

#### Genomic DNA extraction

Genomic DNA was extracted using the boiling method as described by Tadayon . Briefly, about three to four colonies were placed in an Eppendorf tube containing 100 µl of nuclease-free water. The tubes were heated in a water bath at 95 °C for 5 min and then immediately transferred to a freezer at −20 °C for 10 min to lyse the cells. The lysate was centrifuged at 12,000 r.p.m. for 1 min. Using a pipette, 80 µl of the supernatant containing genomic DNA was transferred into a sterile Eppendorf tube. The concentration and purity of the genomic DNA were assessed using a T042-Thermal Scientific NanoDrop Spectrophotometer (Wilmington, DE, USA). The ratio of the A260/A280 was used as a criterion for DNA quality; the extracted DNA was measured, and those having a ratio of 1:8 or below were included for downstream analysis, such as PCR and sequencing. Those that were above the limit ratio were re-extracted. To check for the presence of organic contaminants, the ratio of A260/230A was used; those that fall within the range of 2.0–2.2 were considered good for the downstream analysis. DNA samples that met the quality standards were stored at −20 °C and then used to detect the *invA* gene through PCR. The PCR products were later used for sequencing.

#### Detection of *Salmonella* by PCR

The detection of the *Salmonella* invasive encoding gene was carried out using PCR, targeting the 796 bp region of the gene. The forward primer (*inv-A* 5′ CGGTGGTTTTAAGCGTACTCTT), and the reverse primer (*inv-A* 3′ CGAATATGCTCCACAAGGTTAA), as described by Wang and Yeh [18] were used. AccuPower® PCR PreMix- supplied by Bioneer Corporation (www.bioneer.com) was used to prepare a Master Mix as follows. The PCR Master Mix was prepared by adding 0.5 µl of each forward and reverse primer into the PCR tubes, along with 16 µl of nuclease-free water, and 5 µl of pre-mix. Finally, a DNA template (3 µl) was added, making a total of 25 µl of the PCR reaction mixture. Nuclease-free water and *Salmonella* strains with ATCC 8739 were used as negative and positive controls, respectively.

The PCR amplification process included an initial denaturation step at 95 °C for 5 min, followed by 30 cycles of denaturation at 94 °C for 30 s, annealing at 55 °C for 30 s, extension at 72 °C for 30 s and a final extension at 72 °C for 10 min.

The amplification products were separated by gel electrophoresis using a 1.5% agarose gel stained with ethidium bromide. The PCR products were visualized under UV light using a trans-illumination machine (T2201 *SIGMA* Chemical Company, St. Louis, MO, USA).

#### Sequencing and phylogenetic tree construction

Approximately 15 µl of PCR products that tested positive for *Salmonella* were sent to Macrogen Company for sequencing. DNA sequencing was performed using Sanger sequencing technology in two directions by using forward and reverse primers. BigDye Terminator v3.1 (Applied Biosystems, USA) in the Genetic Analyzer was used. The raw sequences were manually inspected for chimeric sequences. Forward and reverse reads were assembled to generate consensus sequences using Geneious Prime 2024.0.5 (https://www.geneious.com/). The consensus sequences were blasted in the NCBI GenBank (https://blast.ncbi.nlm.nih.gov/Blast.cgi) for comparing similarities. The consensus sequences were also submitted to GenBank via gb-admin@ncbi.nlm.nih.gov to obtain accession numbers. Reference sequences of *Salmonella enterica* subsp. *enterica* serovars were downloaded from GenBank for phylogenetic comparison (Table S1). Sequence alignment was performed using MAFFT version 7 [19], and the aligned sequences were manually trimmed at the ends to ensure consistency. A maximum likelihood phylogenetic tree was inferred using RAxML v.8.2.12 [20] with 1,000 bootstrap replications. The general time-reversible model with gamma-distributed rate variation among sites (GTR+Γ) was selected as the nucleotide substitution model for the analysis. Furthermore, *Salmonella bongori* was downloaded from GenBank and used as an outgroup sequence for comparative purposes as described in Rivera, Pavon and Rahman [21].

### Antimicrobial susceptibility testing

Antimicrobial susceptibility testing was conducted using the Kirby–Bauer disc diffusion method on Mueller–Hinton Agar (MHA) (Oxoid, Basingstoke, UK), following the Clinical and Laboratory Standards Institute guidelines [1]. The *Salmonella* isolates were tested against the antibiotics listed in Table 1.

**Table 1.** Antibiotic concentration and interpretation breakpoints [1]

Antimicrobial agent (code)	Disc concn (µg)	Breakpoint (mm)		
		Sensitive (S)	Intermediate (I)	Resistant (R)
AMP	10	≥17	14–16	≤13
AT	15	≥13	–	≤12
CRO	30	≥23	20–22	≤19
CIP	30	≥26	22–25	≤21
GEN	10	≥15	13–14	≤12
SXT	30	≥16	11–15	≤10
TE	30	≥15	12–14	≤11

Interpretation of antimicrobial susceptibility was conducted according to Clinical and Laboratory Standards Institute (CLSI) guidelines [1]. Disc concentrations and corresponding zone diameter breakpoints are expressed in millimeters (mm). “S” = Sensitive, “I” = Intermediate, “R” = Resistant. “-” indicates that no intermediate breakpoint is defined.

* Interpretation criteria based on Clinical and Laboratory Standards Institute guidelines [1]. Zone diameters are in millimeters (mm). “S” = Sensitive, “I” = Intermediate, “R” = Resistant. A dash (–) indicates no intermediate category defined.

AMP, ampicillin; AT, azithromycin; CIP, ciprofloxacin; CRO, chloramphenicol; GEN, gentamycin; SXT, trimethoprim/sulfamethoxazole; TE, tetracycline.

These antibiotics were selected based on their clinical relevance and usefulness in animal production as outlined by the World Health Organization [22].

Pure colonies of *Salmonella* isolates confirmed by biochemical testing and PCR were subjected to susceptibility tests. Overnight pure culture of *Salmonella* was emulsified into sterile saline to achieve turbidity equivalent to the 0.5 McFarland standard, ~1.5×10^8^ c.f.u. ml^−1^. The suspensions were evenly spread on MHA plates using sterile cotton swabs, followed by aerobic incubation at 37 °C for 16–18 h.

Zones of inhibition were measured using a Vernier calliper, and the results were interpreted according to the 2022 CLSI guidelines. *Salmonella* strain with ATCC 8739 was used as the control strain. A strain was classified as MDR if it exhibited resistance to at least three different antibiotic classes [1].

### Data analysis

Data were entered into Microsoft Excel for initial organization and processing. Descriptive statistics for social demographics variables were then analysed using the Statistical Package for Social Sciences version 20 (SPSS version 20).

## Results

### Social demographic information

The social demographic analysis of 224 poultry farmers in Dar es Salaam revealed that the majority were aged 45–54 years (23.2%), while a smaller proportion (6%) were aged above 65. The samples included slightly more females (51.8%) than males (48.2%), and it was observed that most of the participants were married (60.3%), while 30.8% were single and 8.9% were widowed. The participants were predominantly from Temeke districts, 43.3% (*n*=97); compared to Ilala, 33.48% (*n*=75); and Kigamboni, 23.2% (*n*=52). Surprisingly, the majority of farmers raising chickens possessed at least a secondary education, with 89.3% (*n*=200) being business owners, a higher percentage than the 10.7% of 224 farmers who were employed, as shown in Table 2.

**Table 2.** Social demographic information showing frequencies of farmers involved in poultry farming activities in three districts in Dar es Salaam, Tanzania

Social demographic	Frequency (n)	Per cent (%)
**Age range**		
Age 15–24	44	19.6
Age 25–34	40	17.9
Age 35–44	49	21.9
Age 45–54	52	23.2
Age 55–64	25	11.2
Age >65	14	6.3
**Gender**		
Female	116	51.8
Male	108	48.2
**Marital status**		
Married	135	60.3
Single	69	30.8
Widow	20	8.9
**Districts**		
Ilala	75	33.5
Kigamboni	52	23.2
Temeke	97	43.3
**Level of education**		
College and above	42	18.8
Primary school	84	37.5
Secondary school	98	43.8
**Occupation**		
Businessman/woman	200	89.3
Occupation formal employment	24	10.7
**Total**	**224**	**100**

The table summarizes socio-demographic characteristics of 224 farmers engaged in poultry farming across three districts in Dar es Salaam, Tanzania. Percentages are based on total respondents (n = 224).

### *Salmonella* isolation and identification

Of the 796 faecal samples cultured in BPW, RVS, and on XLD agar, 24.6% (*n*=196) were identified as presumptive *Salmonella* spp. All the presumptive *Salmonella* isolates (*n*=196) were subjected to a battery of biochemical tests, including their reactions to the indole test, TSI agar, urease, motility and l-lysine decarboxylase (Table 3). All isolates showing positive reactions on TSI positivity (74%, 145), l-lysine decarboxylase (62.8%, 123) and motility (99.5%, 195) but negative reaction on indole (99.5%, 195) and urease negativity (65.8%, 129), which were regarded as belonging to *Salmonella* spp. as these characteristics are essential for differentiation of *Salmonella* spp. from other enteric bacteria. Based on the overall biochemical profile, 48 (24.5%) isolates were confirmed as *Salmonella* spp., while 148 isolates (75.5%) were classified as non-*Salmonella*. The frequency distribution is summarized in Table 3.

**Table 3.** Frequency distribution of *Salmonella* isolates grown in XLD media for the biochemical tests

Biochemical test	Positive *N* (%)	Negative *N* (%)	Total *N* (%)
Indole	1 (0.5)	195 (99.5)	196 (100)
TSI	145 (74)	51 (26)	196 (100)
Urease	67 (34.2)	129 (65.8)	196 (100)
Motility (SIM)	195 (99.5)	1 (0.5)	196 (100)
l-Lysin decarboxylase	123 (62.8)	73 (37.2)	196 (100)
**Overall results**	**48** (**24.5**)	**148** (**75.5**)	**196 (100**)

The table presents the biochemical test outcomes for 196 presumptive *Salmonella* isolates cultured on XLD agar. Results are expressed as the number and percentage of isolates testing positive or negative for each biochemical marker. Percentages are based on the total number of isolates tested (n = 196).

SIM, sulfur indole motility; TSI, triple sugar ion.

#### Prevalence of *Salmonella* spp. in poultry faecal droppings

A total of 796 poultry faecal samples were collected from 224 poultry farms in three districts (Kigamboni, Ilala and Temeke) in Dar es Salaam, Tanzania. Twenty-four per cent (48 out of 196) were confirmed as *Salmonella* spp. based on the combined biochemical test results, which is the prevalence of 6% (48 out of 796) was observed from the initial faecal droppings.

#### Molecular detection of *Salmonella* spp.

The PCR assay was carried out in this study for the detection of the *Salmonella invA* gene from the 48 *Salmonella*-positive samples. Due to financial constraints, 28 samples (58.33%) were randomly selected for PCR, ensuring a representative distribution across the districts to confirm the presence of *Salmonella* isolates. A blinded third party who was not involved in the isolation and identification was used to select the samples.

**Fig. 2.**
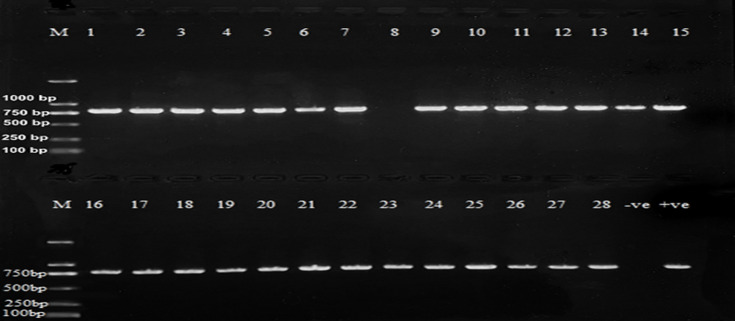
Amplified *InvA* gene, where M is a DNA molecular marker (1 kbp), lanes 1–28 are test samples and lanes -ve and +ve are for positive and negative controls, respectively.

The PCR results revealed that 96.43% (*n*=27) of the selected samples tested positive for the *invA* gene, while 3.57% (*n*=1) were negative for the *invA* gene. The result is as illustrated in Fig. 2.

#### Sequence and phylogenetic analysis

**Fig. 3.**
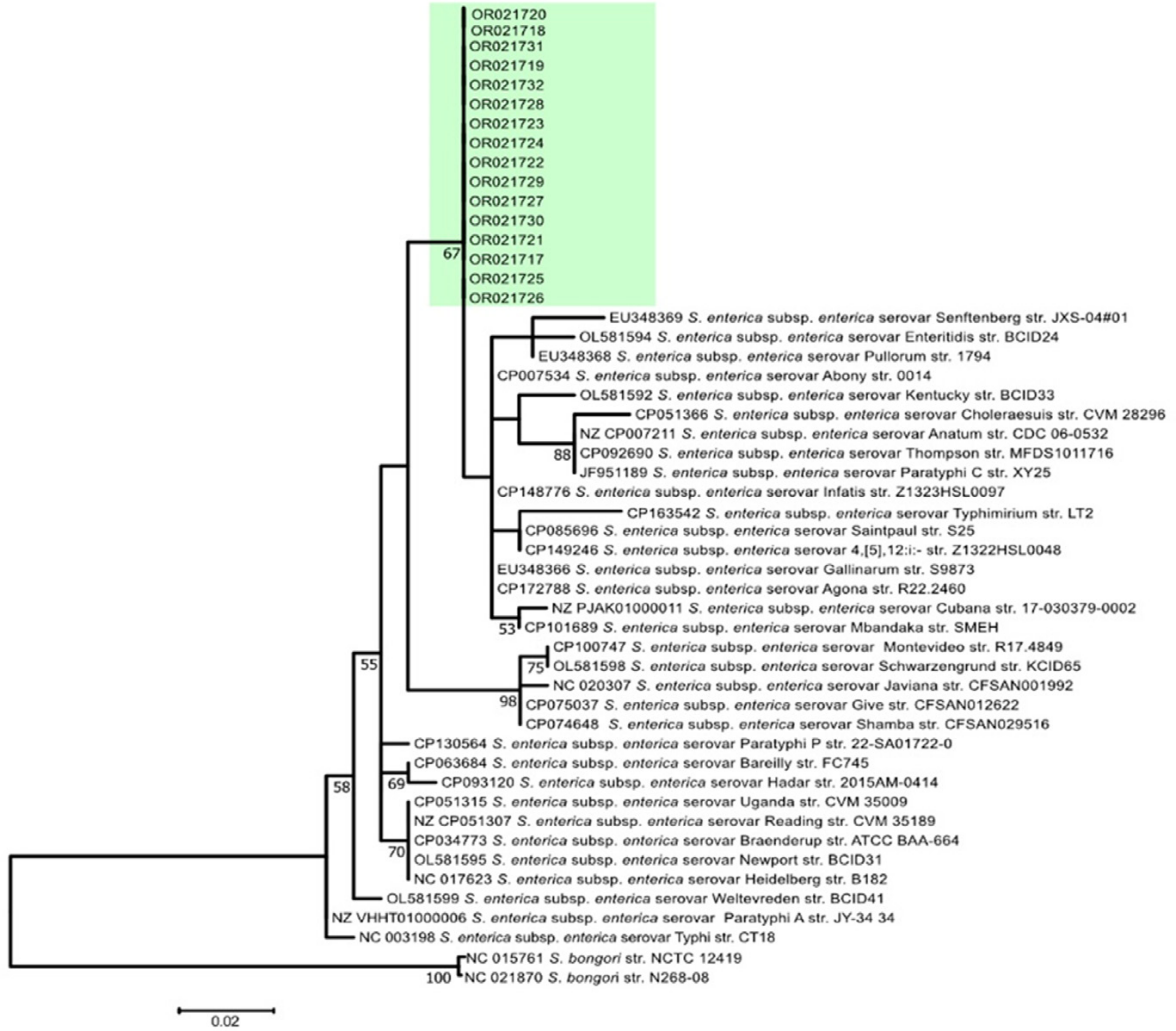
Phylogenetic tree analysis indicates 16 sequences of *Salmonella* isolates from poultry farms, 32 isolates retrieved from GenBank and 2 outgroup species.

The phylogenetic analysis on the *Salmonella* subspecies based on the *invA* gene was performed using the sixteen sequences of *Salmonella* isolates with accession numbers OR021717-OR021739 obtained from this study and 32 sequences retrieved from GenBank. The phylogenetic tree (Fig. 3) revealed that all the 16 sequences clustered in a close relationship with * S. enterica* with accession numbers EU348369 strains Senftenberg isolated from China, OL581594 *Salmonella* Newport isolated from China and EU348368 *Salmonella* Pullorum isolates from China. Other sequences diverged more distantly; these are *Salmonella* Abony with accession number CP007541 and *Salmonella* Kentucky with accession number OL581592. The tree also included an outgroup species, *S. bongori,* which was downloaded from GenBank with accession numbers NC015761 and NC021870 *S. bongori*. The cluster highlighted the genetic relatedness between the *Salmonella* isolates from poultry from this study and *S. enterica* from other sources.

### Antimicrobial susceptibility testing

Table 4 represents the antimicrobial susceptibility profiles of bacterial isolates against seven antibiotics, revealing significant variability in effectiveness. Ampicillin (AMP) and tetracycline (TE) were the least effective, with high resistance rates of 92.9% (*n*=26) and 71.43% (*n*=20), respectively, and minimal or no sensitivity. Ciprofloxacin (CIP) also showed limited efficacy, with no fully sensitive isolates, 42.9% (*n*=12) resistance and 57.1% (*n*=16) intermediate response. Trimethoprine/Sulfamethoxazole (SXT) and gentamycin (GEN) demonstrated moderate effectiveness, with 57.14% (*n*=16) and 64.3% (*n*=18) sensitivity, respectively, although both had notable resistance rates. Azithromycin (AT) performed better, with 71.4% (*n*=20) sensitivity and only 28.6% (*n*=8) resistance. Chloramphenicol (CRO) emerged as the most effective antibiotic, achieving 100% (*n*=28) sensitivity with no intermediate or resistant cases.

**Table 4.** Antibiotic sensitivity patterns of *Salmonella* isolates from poultry farms

Antimicrobial agent	Sensitive *N* (%)	Intermediate *N* (%)	Resistant *N* (%)
AMP	0 (0%)	2 (7.1%)	26 (92.9%)
AT	20 (71.4%)	0 (0%)	8 (28.6%)
CRO	28 (100%)	0 (0%)	0 (0%)
CIP	0 (0%)	16 (57.1%)	12 (42.9%)
GEN	18 (64.3%)	0 (0%)	10 (35.7%)
SXT	16 (57.14%)	0 (0.0%)	12 (42.85%)
TE	8 (31%)	0 (0.0%)	20 (71.43%)

Antibiotic susceptibility testing of *Salmonella* isolates was performed using the disc diffusion method in accordance with CLSI (2022) [1] guidelines. Results are expressed as the number and percentage of isolates classified as Sensitive (S), Intermediate (I), or Resistant (R). Percentages are based on the total number of isolates tested for each antibiotic (n = 28).

AMP, ampicilin; AT, azythromycin; CIP, ciprofloxacin; CRO, chloramphenicol; GEN, gentamycin; TE, trimethoprim/sulfamethoxazole.

**Fig. 4.**
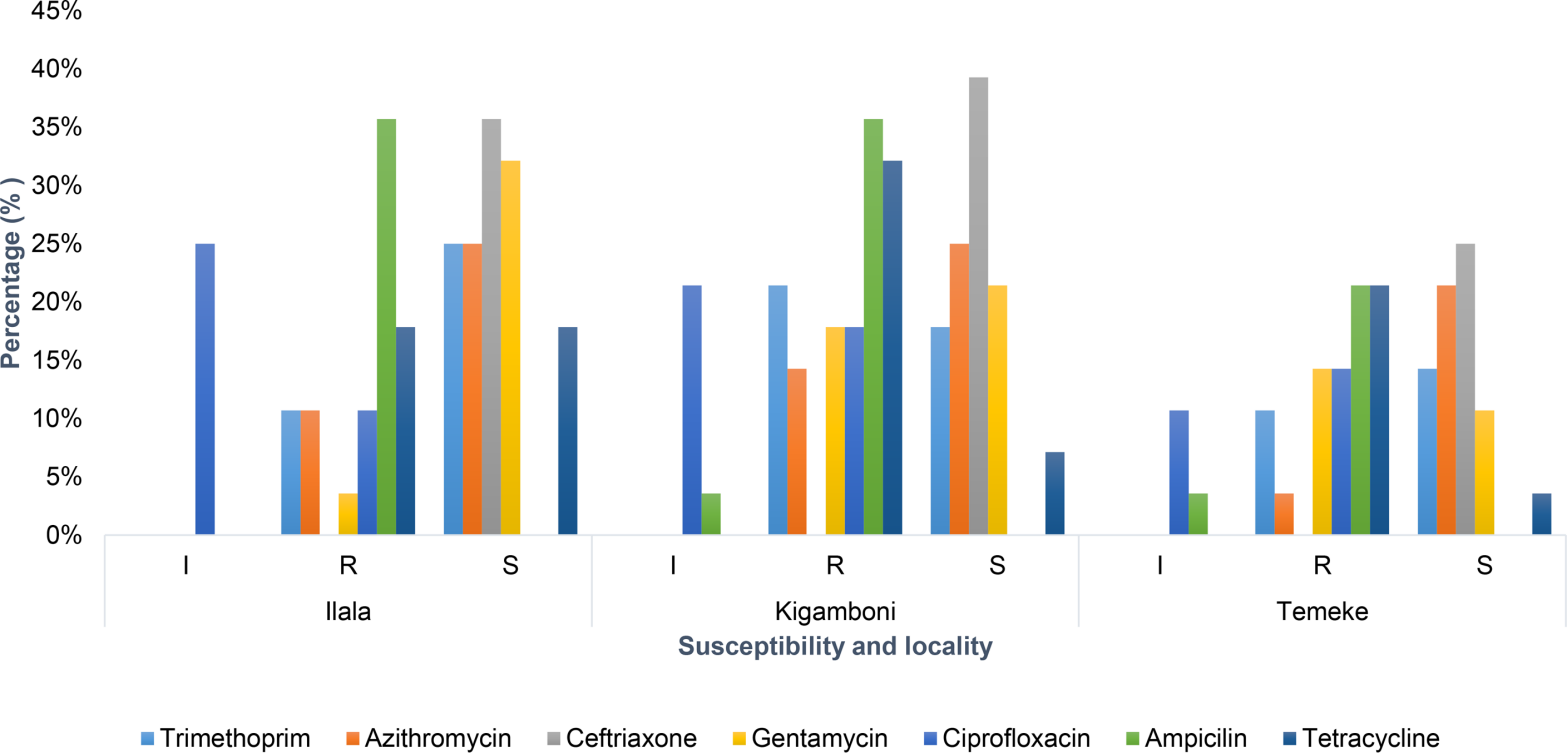
Antibiotic susceptibility patterns of *Salmonella* isolates from poultry farms across three localities (Ilala, Kigamboni and Temeke). The chart shows the percentage distribution of isolates classified as intermediate (I), resistant (R) and susceptible (S) for *Salmonella* isolates.

Fig. 4 shows *Salmonella* isolates from three districts of Ilala, Temeke and Kigamboni and their resistance pattern. Chloramphenicol was effective against all 28 *Salmonella* isolates across the districts, while 26 isolates (92.9%) were resistant to ampicillin and 2 were intermediate.

**Fig. 5.**
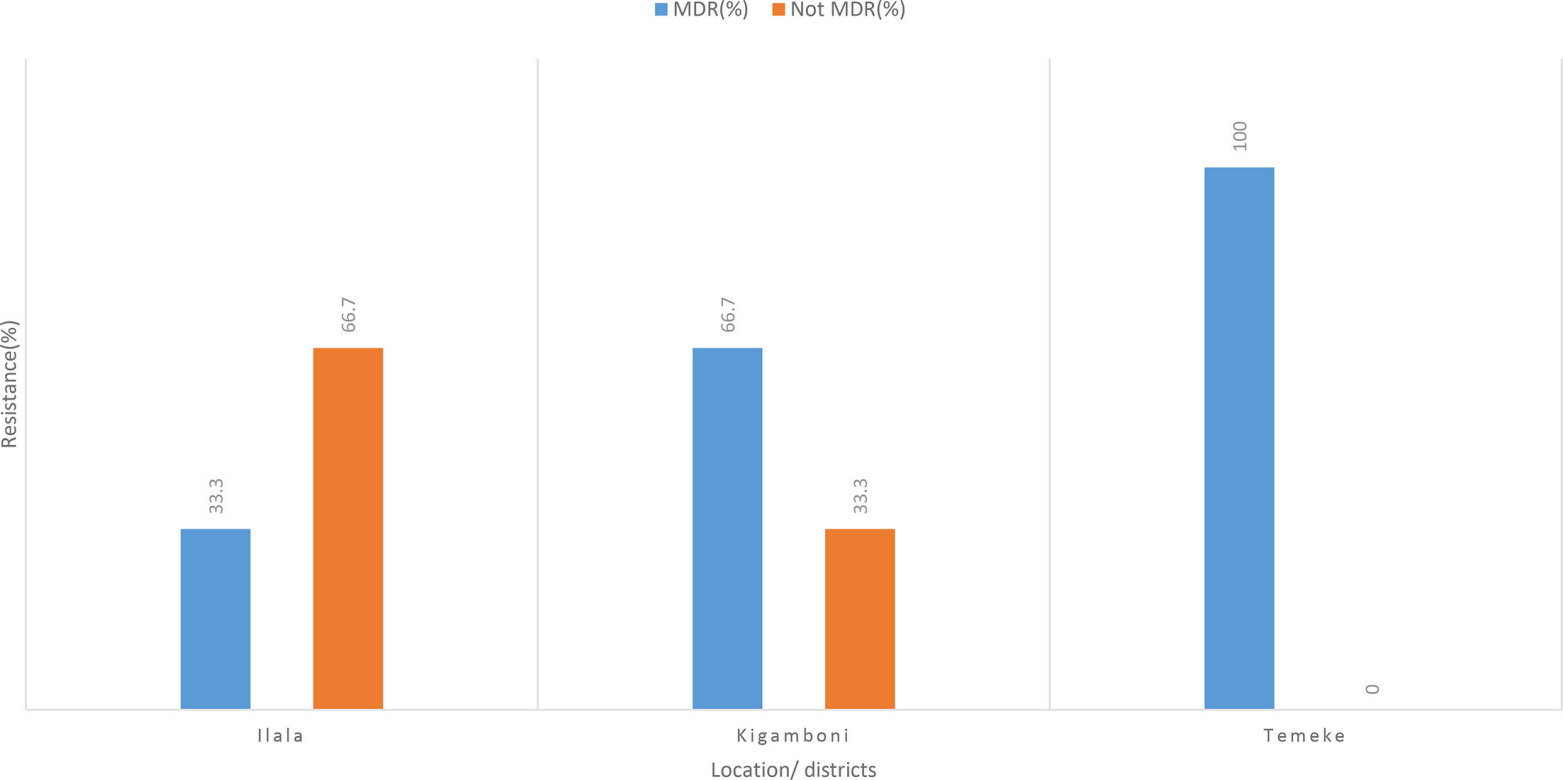
Proportion of *Salmonella* MDR isolates and non-MDR antibiotics in different districts in Dar es Salaam.

The findings further revealed that the proportion of MDR isolates in the three districts was (100%) from Temeke district with (*n*=7) being resistant to more than two classes of antibiotics, followed by Kigamboni (66.7%, *n*=8) and Ilala (33.3%, *n*=3) as shown in Fig. 5

## Discussion

*Salmonella* strains are introduced into poultry flocks through various sources such as contaminated feeds, contaminated water and contaminated environment [7]. Among bacterial and viral infections in chicken farms, *Salmonella* infection has become a significant challenge [23]. Recently, the use of antibiotic drugs in poultry farming has increased, which increases the risk of the rise of AMR, which poses a threat to public health [24]. Despite the rampant use of antibiotics, this study managed to isolate *Salmonella*, indicating that the chicken in the selected farms was infected with *Salmonella*. The antibiotics are used not only for the treatment of infection in animals, but also they are used in improving feed efficiency, promoting growth and enhancing productivity [25]. However, rampant use, misuse and gene transfer are major contributors to the development of antibiotic resistance in bacteria [26]. Antibiotic-resistant bacteria, such as *Salmonella*, can serve as potential vehicles for transferring antibiotic-resistant genes to other bacteria, posing a significant threat to human health [27].

This study detected, characterized and established the antibiotic susceptibility pattern of *Salmonella* isolates from poultry farms in Dar es Salaam, Tanzania, suggesting that chickens and their droppings contain MDR *Salmonella* strains, which might contribute significantly to the infection and antibiotic resistance transfer from poultry to the environment and humans. Our study assessed the social demographic information, antibiotic sensitivity and resistance patterns of the isolates. Findings from this study suggest that poultry are significant reservoirs for multidrug-resistant strains, necessitating a detailed analysis of these isolates and their multidrug resistance patterns, which concur with findings from other studies by Foley *et al*. [7]

### Social demographic information

From this study, it was observed that the majority of the respondents were female (51.8%). This reflects the fact that poultry production has been largely a gender-based occupation in many resource-limited settings, where it serves as a means of income for women. This finding is consistent with other findings reported by [28,30]. Most of the respondents were aged 45–54 years (23.2%), followed by 35–44 years (21.9%). These age groups are likely engaged in poultry farming as an alternative livelihood or as a means to support their families, as younger individuals may still be pursuing education, while those nearing retirement may explore income generation activities [31]. This could be due to the reported increase in demand for proteinaceous products such as meat and eggs in the population, as reported by Ringo [32].

Married individuals accounted for 60.3% of the respondents, indicating that poultry farming plays a significant role in family income and empowerment. Similar findings have been reported [33]. Regarding education levels, most poultry keepers had a secondary education (43.8%) with limited formal training in poultry keeping management and proper use of antibiotics. This gap may lead to improper use of antimicrobials in poultry keeping management. In Tanzania, the secondary level does not have courses that directly explain the AMU and the effects of AMR. These results call for more alarm to the issue of knowledge, attitudes and practices among these emerging poultry keepers. Similar results were reported by Kimera *et al*. [34]. Additionally, the findings revealed that 89.3% engaged fully in the poultry-keeping business. This indicates that poultry farming is a crucial livelihood strategy, particularly for individuals who may lack formal employment opportunities.

### Prevalence, isolation and identification of *Salmonella* spp.

Conventional microbiological methods were used for the isolation and preliminary identification of *Salmonella* isolates before PCR confirmation. The finding revealed that of 796 samples cultured in XLD, only 24.5% (*n*=196) were presumptive *Salmonella* spp. Biochemical characteristics revealed that 24.48% (*n*=48) of isolates were positive for *Salmonella*. The *Salmonella* positivity rate, determined through conventional microbiology methods, was 6% (48 out of 796 collected samples). These findings show a moderate presence of *Salmonella* in the population samples, further indicating its significance as a potential public health concern. Furthermore, the findings indicate the importance of using conventional microbiological techniques in the isolation and identification of bacterial pathogens and hence concur with a previous report by [35]. Additionally, among the media used, SIM medium yielded the highest presumptive positive rate (99.5%), followed by TSI 74%, and indole positive 0.5% indicating its specificity and reliability in identifying *Salmonella*.

The study emphasizes enhanced surveillance and control to mitigate *Salmonella* transmission, particularly in environments contaminated with faecal droppings. Moreover, the prevalence of 6% is similar to other findings, although some variations may arise due to different methods of sampling, geographical locations and environmental factors as well as sample type [36], [37]. PCR was used to confirm the presence of Salmonella spp. by targeting the invA gene. The results indicated that 93.3% of the biochemically positive isolates were confirmed as *Salmonella* by PCR, closely aligning with the biochemical test results. This suggests that in resource-limited countries, conventional methods used in microbiology may remain valuable for *Salmonella* detection, as previously suggested by Andrews *et al*. [38]. To provide a comprehensive understanding of *Salmonella* prevalence in environmental or clinical samples, there is a need to use integrated approaches by combining culture, biochemical and advanced tools such as molecular techniques.

### Phylogenetic tree analysis

From phylogenetic tree analysis, it was observed that all 16 sequences of *Salmonella* spp. (OR021717-OR021739) isolated from Tanzania clustered together and shared a recent common ancestor with a bootstrap value of 67. The clustering shows that there is limited genetic diversity of *Salmonella enterica* strains in Dar es Salaam, which could be due to similar environmental conditions, such as using contaminated poultry feeds, and poultry management as a whole could be the factors that contributed to these results.

The other reason for such closeness and similarities might be due to the sample source since all the samples came from poultry faecal droppings. The genetic relationship and diversity of the 16 sequences and those downloaded from GenBank show that sequence EU348369 was the most closely related to the 16 sequences from this study, which were isolated from a foodborne disease outbreak in Shenzhen, Guangdong province in China [39]. These results suggest that this sequence shares a common ancestor with the 16 isolates from Tanzania. This might be due to international trade of poultry and poultry products, international travel and migration, a globalized feed supply chain, genetically related poultry lines, and others, which contribute to the genetic similarity and close relatedness of the *Salmonella* strains in those isolates, indicating the global dissemination of *Salmonella* strains. Other sequences that were closely related to each other are OL581594 (*S. enterica* serovar Enteritidis) and EU348368 (*S. enterica* serovar Pullorum), suggesting a potential evolutionary link between the sixteen isolates and those from GenBank representing a variant or closely related lineage of *Salmonella enterica* serovars such as * S.* Enteritidis or *S.* Pullorum. Other sequences such as CP007541 (*S. enterica* Abony) and OL581592 (*S. enterica* Kentucky) represented more genetically distant serovars, and the broader tree structure highlighted their divergence from more distant *S. enterica* lineages. Therefore, from this study, the clustering of *Salmonella* isolates from Tanzania and their close relationship with strains from Africa, East Africa, globally highlight the need for strong biosecurity, improved surveillance and enhanced food safety measures. Collaborative efforts at national, regional and global levels are crucial to prevent *Salmonella* infections and address antimicrobial resistance challenges. Public awareness and education remain key to ensuring consumer safety and reducing the burden of *Salmonella*-related diseases.

### Antimicrobial susceptibility test

There are several causes behind the rise of antibiotic resistance in chicken farming operations. In this study, the findings revealed that commonly used antibiotics such as ampicillin and tetracycline, which scored 92.9% and 69%, respectively, showed high resistance rates among the isolates. This resistance is likely due to the misuse of antibiotics, including their use as growth promoters and for disease prevention [40]. Findings of this study are consistent with previous studies conducted in Tanzania and neighbouring countries such as Kenya and Uganda [41, 42]. Moreover, the observed resistance may be because there are unwarranted practices regarding the use of antibiotics among poultry farmers as a sole alternative in managing poultry disease outbreaks and transmission, as previously reported [43]. In the majority of low- and middle-income countries including Tanzania, tetracycline, aminoglycosides and penicillin have been reported as the most widely used class of antibiotics in the treatment of bacterial diseases in animals [44].

The study also found that chloramphenicol and gentamicin, which scored 100% and 64.3% respectively, were effective against *Salmonella* isolates, likely due to their limited use in poultry farming. Resistance to ciprofloxacin (42.9%) and its intermediate susceptibility (57.1%) highlight the need for close monitoring of fluoroquinolone use. Additionally, a farmer may decide to switch up the medications they use to treat their chickens to prevent loss. Alarmingly, in the course of this study, it was discovered that certain farmers use human medications, particularly the combination of ampicillin and cloxacillin, whenever they do not notice improvements in the way their poultry are treated by veterinary medications. These farmers believe that using human medications produces better results than using veterinary medications, so they are beginning to lose faith in veterinary medications and have chosen to switch to using human medications instead when treating the flu. This behaviour may contribute to high resistance of *Salmonella* not only to the poultry sector but also to humans, especially when farmers engage in the rampant use of human antibiotics in poultry farming [45]. Based on findings from this study, we proposed that the Tanzanian Medicines and Medical Devices Authorities such as TMDA, in partnership with the Tanzania Veterinary Laboratory Association (TVLA), do comprehensive research on the subject and provide education to the farmers about the significance of using antibiotics appropriately in their farms.

### Multidrug resistance patterns

The study revealed that (64.3%, *n*=18) *Salmonella* isolates were resistant to more than two antibiotic classes, indicating a high prevalence of MDR. This was seen in all districts such as Temeke (100%, *n*=7), Kigamboni (66.7%, *n*=8) and Ilala (33.3%, *n*=3), which were resistant to more than two antibiotics. Additionally, 35.3% of the isolates were resistant to fewer than two antibiotics (non-MDR), showing resistance to fewer than two antibiotics. This is an indication of a growing AMR problem in poultry production that requires a multi-sectoral call for action at all levels. These findings highlight the urgent need for awareness campaigns, improved biosecurity and alternative strategies to manage poultry diseases without relying heavily on antibiotics.

## Limitations of the study

### Cross-sectional design

The study only provides a snapshot of the prevalence and resistance patterns of *Salmonella* because it did not cover seasonal trends, longitudinal resistance development or causal relationships of the pathogen among poultry farmers.

### Potential bias in the sampling method

It is likely to be biassed when snowball sampling is used, especially in the selection of the farmers who participated in the research, and sometimes having farmers who share similar farming practices, similar geographical characteristics and only locate farmers who they know and leaving those they do not know, which may lead to limiting the diversity of the sample population.

### The limited scope of antimicrobial testing

The study used seven classes of antibiotics, while there are more than seven classes of antibiotics that are used in veterinary medicines for the treatment and management of *Salmonella* infections in poultry farming.

### Sequencing of the resistance gene

In this study, the resistance gene (genotypic traits) was not considered; only phenotypic traits were considered. Therefore, it might be possible that the number of resistant isolates could be more genotypic compared to phenotypic.

## Conclusion

The high level of antibiotic resistance found in this study has serious implications, which include reduced effectiveness of commonly used antibiotics, the presence of antimicrobial residues in poultry products and the potential spread of resistant bacteria to humans and the environment. Immediate actions are needed to address these challenges, such as limiting the use of antibiotics in poultry production systems at the farm level and improving hygienic procedures at poultry houses. Prudent use of antibiotics and biosecurity policies should also be advocated to govern disease prevention in poultry production systems as a sustainable solution to the ever-growing problem of AMR.

## Supplementary material

10.1099/acmi.0.000879.v5 Uncited Table S1.

## Abbreviations

AMP: ampicillin
AT: azithromycin
BPW: buffered peptone water
CIP: ciprofloxacin
CRO: chloramphenicol
GEN: gentamycin
MDR: multidrug-resistant
MHA: Mueller–Hinton agar
RVS: Rappaport Vassiliadis medium
SIM: sulphide indole motility
SXT: trimethoprim/sulfamethoxazole
TE: tetracycline
TMDA: Tanzania Medicines and Medical Devices Authority
TSI: triple sugar iron
XLD: xylose lysine deoxycholate
