# Supplementary material for: Detection and antibiotic resistance of Salmonella isolates from selected poultry farms in Dar es Salaam, Tanzania
Source: Access Microbiol. 2025 May 21;7(5):000879.v5. doi: 10.1099/acmi.0.000879.v5 (PMC12095867; doi:10.1099/acmi.0.000879.v5)
Supplement: Uncited Table S1. [file acmi-7-00879-s001.pdf]

Supplementary table 1. List of *Salmonella* species used for phylogenetic analysis

Species	Subspecies	Serovar	Strain	Source	Country	Accession number
S. enterica	*enterica*	Kentucky	BCID33	Chicken carcass	Indonesia	OL581592
S. enterica	*enterica*	Typhimurium	KCID11	Chicken carcass	Indonesia	OL581591
S. enterica	*enterica*	Typhimurium	BCID12	Chicken carcass	Indonesia	OL581590
S. enterica	*enterica*	Pullorum	1794	-	China	EU348368
S. enterica	*enterica*	Gallinarum	S9873	-	China	EU348366
S. enterica	*enterica*	Saintpaul	S25	Human stool	China	CP085696
S. enterica	*enterica*	4,[5],12:i:-	Z1322HSL0048	Human	South Korea	CP149246
S. enterica	*enterica*	Agona	R22.2460	-	Taiwan	CP172788
S. enterica	*enterica*	Infantis	Z1323HSL0097	Human	South Korea	CP148776
S. enterica	*enterica*	Abony	0014	-	USA	CP007534
S. enterica	*enterica*	Cubana	17-030379-0002	Dry chia seeds	Canada	NZ_PJAK01000011
S. enterica	*enterica*	Mbandaka	SMEH	Human	China	CP101689
S. enterica	*enterica*	Paratyphi C	XY25	Goose anal swab	China	JF951189
S. enterica	*enterica*	Thompson	MFDS1011716	Cookware	South Korea	CP092690
S. enterica	*enterica*	Anatum	CDC 06-0532	Human stool	USA	NZ_CP007211
S. enterica	*enterica*	Choleraesuis	CVM 28296	Pig	USA	CP051366
S. enterica	*enterica*	Typhi	CT18	Human blood	Vietnam	NC 003198
S. enterica	*enterica*	Paratyphi A	JY-34 34	Human blood	China	NZ_VHHT01000006
S. enterica	*enterica*	Weltevreden	BCID41	Chicken carcass	Indonesia	OL581599
S. enterica	*enterica*	Newport	BCID31	Chicken carcass	Indonesia	OL581595
S. enterica	*enterica*	Reading	CVM 35189	Cattle	USA	NZ_CP051307
S. enterica	*enterica*	Heidelberg	B182	Cattle feces	France	NC_017623
S. enterica	*enterica*	Braenderup	ATCC BAA-664	-	-	CP034773
S. enterica	*enterica*	Uganda	35009	Cattle	USA	CP051315
S. enterica	*enterica*	Paratyphi B	22-SA01722-0	Dead mute swan	Germany	CP130564
S. enterica	*enterica*	Hadar	2015AM-0414	Human stool	USA	CP093120
S. enterica	*enterica*	Bareilly	FC745	Human stool	India	CP063684
S. enterica	*enterica*	Senftenberg	JXS-04#01	-	China	EU348369

S. enterica	*enterica*	Typhimurium	LT2	-	-	CP163542
S. enterica	*enterica*	Schwarzengrund	KCID65	Chicken carcass	Indonesia	OL581598
S. enterica	*enterica*	Montevideo	R17.4849	Human stool	Taiwan	CP100747
S. enterica	*enterica*	Shamba	CFSAN029516	Raw macadamia nut	USA	CP074648
S. enterica	*enterica*	Give	CFSAN012622	Ground red chili pepper	Thailand	CP075037
S. enterica	*enterica*	Javiana	CFSAN001992	-	USA	NC_020307
S. bongori	-	-	NCTC 12419	-	-	NC_015761
S. bongori	-	-	N268-08	-	Switzerland	NC_021870
